# Disparities Between Rural and Urban Patients With Prostate Cancer in Nebraska

**DOI:** 10.1002/cam4.70812

**Published:** 2025-03-24

**Authors:** Cassie Liu, Kaeli K. Samson, Oleg Shats, Raymond Bergan

**Affiliations:** ^1^ Eppley Institute for Research in Cancer and Allied Diseases, Fred & Pamela Buffett Cancer Center University of Nebraska Medical Center Omaha Nebraska USA; ^2^ Division of Oncology and Hematology, Department of Internal Medicine, Fred & Pamela Buffett Cancer Center University of Nebraska Medical Center Omaha Nebraska USA; ^3^ Department of Biostatistics, College of Public Health University of Nebraska Medical Center Omaha Nebraska USA

**Keywords:** age of death, age of diagnosis, cancer disparity, iCaRe2, Nebraska, prostate cancer, rural, rural–urban disparity, urban

## Abstract

**Introduction:**

Studies focused on rural–urban disparities in patients with prostate cancer have demonstrated minimal differences in incidence and overall survival (OS). However, available data are limited, especially in understudied geographic locations. In this study, we investigated additional measures of potential cancer disparity and focused on examining rural–urban prostate cancer disparity in Nebraska residents.

**Methods:**

Patients diagnosed with prostate cancer from 1991 to 2023 living in Nebraska were identified in the integrated Cancer Repository for Cancer Research (iCaRe2) and categorized as rural and urban by rural–urban commuting area (RUCA) codes.

**Results:**

Results are presented as mean ± standard deviation. The iCaRe2 patient registry contained data on 765 men with prostate cancer living in Nebraska, 621 (81.2%) of whom were urban residents and 144 (18.8%) of whom were rural residents. Rural residents were diagnosed with prostate cancer 3.1 years younger than urban residents (rural: 65.6 ± 8.21 years, urban: 68.7 ± 9.08 years, *p* < 0.001). Rural residents died of prostate cancer 4.2 years younger than urban residents (rural: 72.9 ± 9.75 years, urban: 77.1 ± 8.85 years, *p* < 0.001). Analyses of Gleason score and AJCC stage did not reveal statistically significant differences between rural and urban residents. OS was similar between rural and urban men in Nebraska with prostate cancer, congruent with currently published literature.

**Conclusions:**

Our analysis demonstrates that rural patients in Nebraska are diagnosed and die with prostate cancer at younger ages compared to urban patients. Our findings offer strategies to better define and delineate rural–urban cancer disparity and support future, more robust investigations to consider novel approaches to determining disparities in cancer disease course.

**Abbreviations**
AJCC: American Joint Committee on Cancer
CI: confidence interval
GGG: Gleason Grade Groups
GUCARE: Genitourinary Cancer Registry
HPV: human papillomavirus
HR: Hazard Ratio
iCaRe2: integrated Cancer Repository for Cancer Research
ISUP: International Society of Urologic Pathology
OS: overall survival
RUCA: rural–urban commuting area
SD: standard deviation
SEER: Surveillance, Epidemiology, and End Results
TNM: tumor, node, and metastasis

## 1 Introduction

Rural–urban disparities in US life expectancy have widened since the 1970s, with cancer as one of the leading causes [1, 2]. A recent report using the Surveillance, Epidemiology, and End Results (SEER) database revealed that residing in rural areas in the United States was associated with a 2.4% absolute increase in 5‐year cancer mortality probability [3]. Studies have identified modifiable behaviors, such as tobacco use and human papillomavirus (HPV) infections, as sources of elevated cancer incidence [3, 4, 5] and high resource deprivation as a source of elevated cancer mortality [3] among rural residents.

For prostate cancer, research has extensively focused on disparities in healthcare access between rural and urban communities [6, 7, 8, 9, 10, 11, 12], and incidence, overall survival (OS), and mortality have been the mainstay clinical metrics for investigation. Several former analyses have not found significant modern‐day disparities in incidence and OS [1, 8, 13]. One study even found an increase in incidence in urban populations compared to rural populations, in which the authors cite healthcare access as a likely contributing factor [4, 5]. An analysis of the SEER database demonstrated that rural residents with prostate cancer may experience increased rates of other‐cause mortality while urban residents with prostate cancer may experience marginally higher rates of prostate cancer‐specific mortality [14].

To the best of our knowledge, there are presently no reports that have investigated other dimensions of prostate cancer between rural and urban populations. It is well known that rural communities are exposed to many unique environmental health hazards, and these differences may promote biologic and clinical differences in prostate cancer without altering prostate cancer incidence, OS, and mortality metrics. For instance, several studies identified pesticide exposure to have a positive association with prostate cancer [15, 16]. Moreover, exposure to certain common pesticides has been associated with more aggressive prostate cancer, which studies defined as distant stage, poorly differentiated grade, Gleason score ≥ 7, and/or fatal disease [16, 17, 18].

Almost 35% of Nebraskans are estimated to live in rural areas [19]. This relatively high percentage of rural residents provides an opportunity to examine disparities between rural and urban communities, thereby providing information that can be used to improve health outcomes in both populations. Therefore, we sought to investigate whether rural–urban prostate cancer health disparities exist in Nebraska using the integrated Cancer Repository for Cancer Research (iCaRe2) registry, which has a unique coverage focused on rural areas in the central US great plains region, with about 47% of its registrants coming from Nebraska [20]. Specifically, we examined iCaRe2 for potential differences in prostate cancer‐related metrics, including age of diagnosis, age of death, tumor grade, and clinical stage. We also determined OS in rural and urban populations to compare to published reports.

## 2 Methods

### 2.1 Study Population

The integrated Cancer Repository for Cancer Research (iCaRe2) is a multi‐institutional resource created and maintained by the Fred & Pamela Buffett Cancer Center to collect and manage standardized, multi‐dimensional, longitudinal data and biospecimens on consented adult cancer patients (newly diagnosed and survivors), high‐risk individuals, and healthy controls. The iCaRe2 integrates data provided by subjects via an online patient portal with data from the Electronic Health Records system and other sources. Demographic data, including race and ethnicity, smoking status, education, marital status, household income per year, and employment status, are self‐reported. The iCaRe2 employs a novel metadata‐driven architecture with an Oracle relational database as the backend. It utilizes standard taxonomies to harmonize and transform data from external data sources, thus ensuring computable data availability for research analytics. This study identified a cohort of patients with prostate cancer living in Nebraska by querying the iCaRe2 Genitourinary Cancer Registry (GUCARE).

### 2.2 Variable Definition

Patient zip codes from the iCaRe2 patient registry were mapped to 2010 primary rural–urban commuting area (RUCA) codes [21]. In our analysis, RUCA codes 1–3 were categorized as urban and RUCA codes 4–10 were categorized as rural. Once patients were coded as rural or urban, the following variables were included for analysis: race and ethnicity, smoking status, education, marital status, household income per year, current employment status, age of diagnosis, age of death, Gleason scores and Gleason Grade Groups (GGG), and clinical and pathological tumor, node, and metastasis (TNM) stages. For TNM stage analysis, TNM stages were grouped by T1‐2, T3‐4, N1, and M1. With respect to incomplete stage data, the following assumptions were made: missing N and M data meant N0 and M0, and missing T data was not considered if N1 or M1 existed. To capture the population that underwent curative prostate cancer surgery, metastatic patients were omitted from the analysis of pathological TNM stages. The disaggregation of missing data for TNM staging can be found in Tables S1 and S2. Table S1 lists clinical TNM stage data for 449 urban and 97 rural residents, respectively. Table 2 lists pathological TNM stage data for 124 urban and 43 rural residents, respectively. Since the iCaRe2 registry does not contain isolated PSA levels at the time of diagnosis and many patients lacked pathological TNM staging information, American Joint Committee on Cancer (AJCC) stages were determined using Gleason scores and clinical TNM staging information, and analysis of AJCC stages was restricted to AJCC stages where PSA readings were not needed (stages IIIB–IVB). Patients with missing data were omitted from relevant analyses.

### 2.3 Biostatistical Analyses

Continuous variables were summarized as means ± standard deviations (SD). Associations between categorical variables were assessed using chi‐squared test, or Fisher's exact test when expected cell counts were low. Associations between continuous variables and rural–urban classifications were assessed using the independent samples *t*‐test.

For survival analysis, survival time was calculated using age of diagnosis to age of death if a patient died; all other patients were censored, and their survival time was calculated using age of diagnosis to age last known to be alive. Survival data was plotted using Kaplan–Meier curves. The difference in survival time between rural and urban patients was assessed using a log‐rank test, and median survival time and associated 95% confidence intervals (CIs) were calculated. For an adjusted analysis of survival, a Cox proportional hazards model was performed, which was stratified by clinical TNM stage and included rural–urban status and age. Age was modeled using restricted cubic splines to allow for non‐linearity. The proportional hazards assumptions were assessed, and variables that appeared to violate the assumption were included in an interaction with time in the model. Variables modeled using splines or involved in an interaction with time had hazard ratios calculated at various values (e.g., 25th, 50th, and 75th percentiles) for the variable or follow‐up time.

In instances of missing data, percentages were calculated using the number of patients who had data available as the denominator. All analyses were performed using SAS software version 9.4 (SAS Institute Inc., Cary, North Carolina, USA). All figures, except for the Kaplan–Meier analysis, were generated in GraphPad Prism version 10.2.3 for Windows (GraphPad Software, Boston, Massachusetts, USA).

## 3 Results

### 3.1 Demographic Characteristics of Nebraskans With Prostate Cancer

We identified 765 residents of Nebraska who were diagnosed with prostate cancer from 1991 to 2023, of which 81.2% (*n* = 621) were categorized as urban residents and 18.8% (*n* = 144) as rural residents. Demographic characteristics of the patient populations can be found in Table 1. Urban residents were more racially and ethnically diverse, with 82.4% identifying as non‐Hispanic White men, whereas 98.6% of rural residents identified as non‐Hispanic White men. We evaluated smoking status and found no significant differences between rural and urban residents. Across both populations, the majority of men with prostate cancer were not regular smokers (urban: 56.1%, rural: 66.7%), and among those who reported smoking regularly, the number of smoking years was similar between rural and urban populations. We also analyzed the highest level of education, marital status, household income (per year), and current employment status, which overall revealed no statistically significant difference between groups. Although not statistically significant, a trend emerged for the highest level of education: a greater percentage of rural residents achieved high school graduate (urban: 14.7%, rural: 22.7%), post‐secondary vocational/technical school (urban: 4.7%, rural: 11.4%), and associate degree/some college (urban: 22.9%, rural: 31.8%) as their highest level of education. Urban residents, on the other hand, more often achieved college graduate (urban: 31.8%, rural: 25.0%) and graduate/professional school (urban: 23.5%, rural: 9.1%) as their highest level of education.

**TABLE 1:** Demographic characteristics of Nebraskans with prostate cancer in the iCaRe2 registry.

	Urban (*n* = 621)	Rural (*n* = 144)	*p*
**Race and ethnicity**	*n* = 620	*n* = 144	
American Indian or Alaska Native, not Hispanic/Latino	4 (0.6%)	0 (0.0%)	
Asian, not Hispanic/Latino	6 (1.0%)	0 (0.0%)	
Black/African American	87 (14.0%)	1 (0.7%)	
Not Hispanic/Latino	85 (13.7%)	1 (0.7%)	
Hispanic/Latino	1 (0.2%)	0 (0.0%)	
Unknown	1 (0.2%)	0 (0.0%)	
Multiracial, not Hispanic/Latino	0 (0.0%)	1 (0.7%)	
Native Hawaiian/Pacific Islander, not Hispanic/Latino	2 (0.3%)	0 (0.0%)	
White	521 (84.0%)	142 (98.6%)	
Not Hispanic/Latino	511 (82.4%)	142 (98.6%)	
Hispanic/Latino	5 (0.8%)	0 (0.0%)	
Unknown	5 (0.8%)	0 (0.0%)	
Unknown	1	0	
**Years smoking**	*n* = 157	*n* = 42	0.78^a^
Never smoker/not regular	88 (56.1%)	28 (66.7%)	
Less than 20 years	20 (12.7%)	3 (7.1%)	
20–39.9 years	22 (14.0%)	5 (11.9%)	
40+ years	13 (8.3%)	2 (4.8%)	
Regular smoker, unknown years	14 (8.9%)	4 (9.5%)	
Unknown status	464	102	
**Highest level of education**	*n* = 170	*n* = 44	0.07^a^
Some high school	4 (2.4%)	0 (0.0%)	
High school graduate	25 (14.7%)	10 (22.7%)	
Post‐secondary vocational or technical school	8 (4.7%)	5 (11.4%)	
Associate degree or some college	39 (22.9%)	14 (31.8%)	
College graduate	54 (31.8%)	11 (25.0%)	
Graduate or professional school^b^	40 (23.5%)	4 (9.1%)	
Unknown	451	100	
**Marital status**	*n* = 172	*n* = 44	0.90^a^
Never married	7 (4.1%)	1 (2.3%)	
Married	132 (76.7%)	37 (84.1%)	
Separated	1 (0.6%)	0 (0.0%)	
Divorced	18 (10.5%)	4 (9.1%)	
Widowed	14 (8.1%)	2 (4.5%)	
Unknown	449	100	
**Household income (per year)**	*n* = 144	*n* = 38	0.27^a^
Less than $10,000	4 (2.8%)	0 (0.0%)	
$10,000–24,999	14 (8.7%)	2 (5.3%)	
$25,000–44,999	22 (15.3%)	7 (18.4%)	
$45,000–74,999	38 (26.4%)	10 (26.3%)	
$75,000–100,000	20 (13.9%)	11 (28.9%)	
More than $100,000	46 (31.9%)	8 (21.1%)	
Missing	477	106	
**Current employment status**	*n* = 167	*n* = 42	0.10^a^
Disabled, unable to work	6 (3.6%)	1 (2.4%)	
Employed less than 32 h per week and part‐time student	1 (0.6%)	0 (0.0%)	
Employed less than 32 h per week	3 (1.8%)	3 (7.1%)	
Employed greater than or equal to 32 h per week	42 (25.1%)	16 (38.1%)	
Retired	115 (68.9%)	22 (52.4%)	
Missing	454	102	
**Age of diagnosis (years)**	*n* = 621	*n* = 143	
40–44	1 (0.2%)	1 (0.7%)	
45–49	8 (1.3%)	6 (4.2%)	
50–54	40 (6.4%)	8 (5.6%)	
55–59	58 (9.3%)	17 (11.9%)	
60–64	111 (17.9%)	35 (24.5%)	
65–69	125 (20.1%)	35 (24.5%)	
70–74	119 (19.2%)	22 (15.4%)	
75–79	91 (14.7%)	14 (9.8%)	
80–84	42 (6.8%)	3 (2.1%)	
85–89	22 (3.5%)	2 (1.4%)	
90–94	4 (0.6%)	0 (0.0%)	
Missing	0	1	
*Mean (SD)*	68.7 (9.08)	65.6 (8.21)	< 0.001^c^
**Age of death (years)**	*n* = 426	*n* = 84	
45–49	1 (0.2%)	2 (2.4%)	
50–54	2 (0.5%)	2 (2.4%)	
55–59	16 (3.8%)	3 (3.6%)	
60–64	25 (5.9%)	6 (7.1%)	
65–69	54 (12.7%)	18 (21.4%)	
70–74	66 (15.5%)	16 (19%)	
75–79	82 (19.2%)	18 (21.4%)	
80–84	92 (21.6%)	9 (10.7%)	
85–89	56 (13.1%)	8 (9.5%)	
90–94	32 (7.5%)	2 (2.4%)	
Missing	60	195	
*Mean (SD)*	77.1 (8.85)	72.9 (9.75)	< 0.001^c^

^a^ Fisher's exact *p* value.

^b^ Master's or doctoral degrees.

^c^ Independent samples *t*‐test.

### 3.2 Age of Diagnosis, Age of Death, and Overall Survival

**FIGURE 1:**
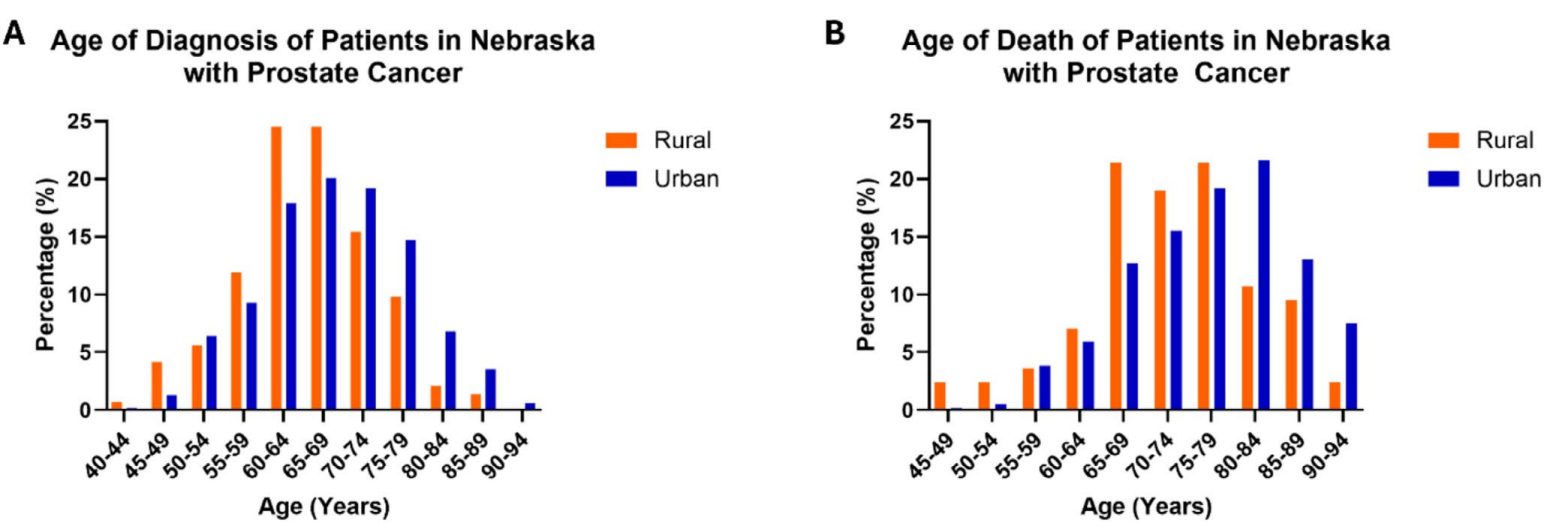
Bar graphs of (A) age of diagnosis and (B) age of death of rural (orange) and urban (blue) patients in Nebraska with prostate cancer. (A) The mean ± SD age of diagnosis for rural patients was 65.6 ± 8.21 years and for urban patients was 68.7 ± 9.08 years (*p* < 0.001). (B) The mean ± SD age of death for rural patients was 72.9 ± 9.75 years and for urban patients was 77.1 ± 8.85 years (*p* < 0.001).

We compared the age of diagnosis (Figure 1A; Table 1) and the age of death (Figure 1B; Table 1) between rural and urban men with prostate cancer in Nebraska. Notably, the distributions for rural men with prostate cancer for both the age of diagnosis and the age of death were shifted to younger ages compared to their urban counterparts. Prostate cancer was diagnosed in rural residents at 65.6 ± 8.21 years and in urban residents at 68.7 ± 9.08 years, with the mean difference of 3.1 years (*p* < 0.001). Even more disparate, rural residents died of prostate cancer at 72.9 ± 9.75 years, while urban residents died of prostate cancer at 77.1 ± 8.85 years, for a difference of 4.2 years (*p* < 0.001).

**FIGURE 2:**
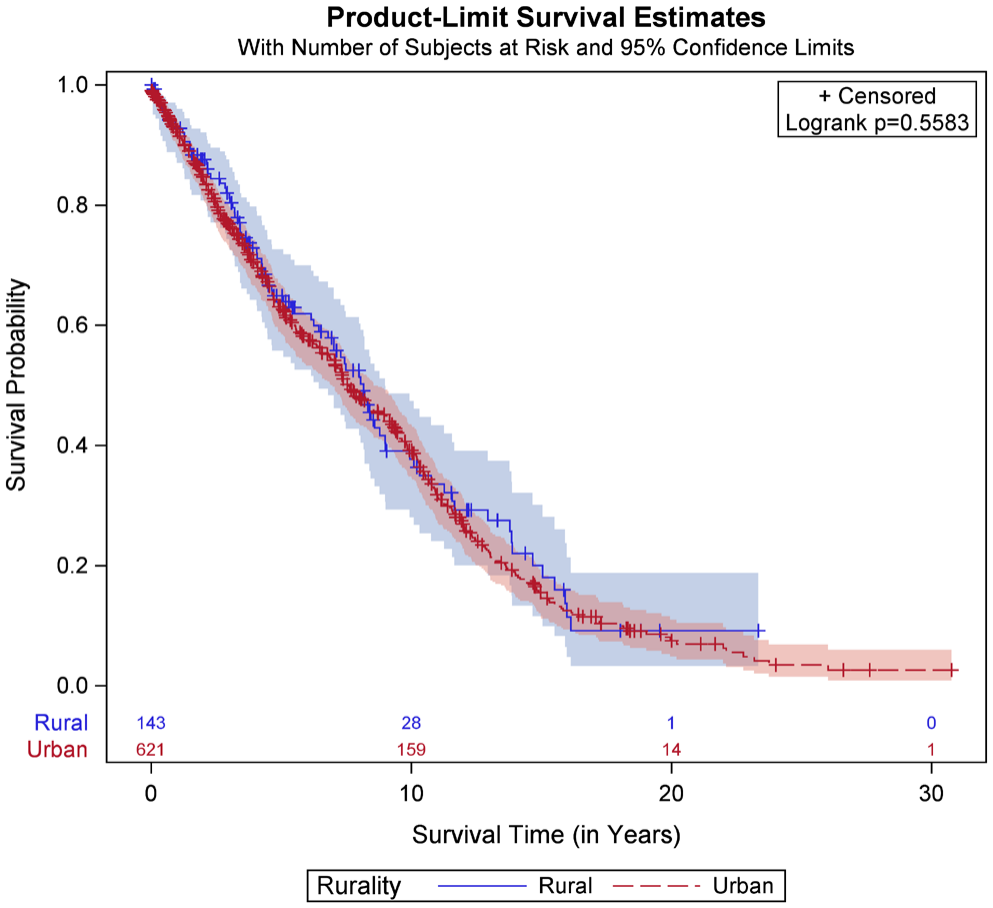
Kaplan–Meier survival graph of overall survival of rural (blue) and urban (red, dashed) patients in Nebraska with prostate cancer (*p* = 0.56). The median survival time for rural patients was 8.17 years (95% CI: 6.45, 8.99) and for urban patients was 7.59 years (95% CI: 6.89, 8.55). At the time of analysis, 41.5% of rural patients and 31.2% of urban patients were alive, and 59.2% of rural patients and 68.8% of urban patients were deceased.

We determined OS (Figure 2) and found no statistically significant difference between rural and urban populations (*p* = 0.56). Rural men with prostate cancer had a median OS of 8.17 years (95% CI: 6.45, 8.99), and urban men with prostate cancer had a median OS of 7.59 years (95% CI: 6.89, 8.55). At the time of analysis, 41.5% of rural patients and 31.2% of urban patients were alive, and 59.2% of rural patients and 68.8% of urban patients were deceased. We did not have enough data on the cause of death to perform a disease‐specific survival analysis.

We performed an additional OS analysis that adjusted for clinical TNM stage through stratification and age by including age in the model with the rural/urban status variable (Table 2). Diagnosis age was modeled using a restricted cubic spline due to its non‐linear relationship with survival, and because the age spline violated the proportional hazards assumption, it was also included in an interaction with time. Overall, there was no statistically significant difference in the adjusted risk of death between rural and urban patients (adjusted HR: 1.11; 95% CI: 0.85, 1.45; *p* = 0.45).

**TABLE 2:** Extended Cox proportional hazards model for survival, stratified by clinical TNM stage.

	Follow‐up time (years)	Adjusted hazard ratio	95% Confidence interval	*p*
Rural–urban status				0.45
Rural		1.11	0.85–1.45	
Urban		1.00	Reference	
Diagnosis age (years)^a^				
62 years old	2	1.00	0.97–1.03	
	5	1.02	1.00–1.04	
	10	1.05	1.02–1.08	
68 years old	2	0.98	0.93–1.02	
	5	0.99	0.96–1.03	
	10	1.02	0.97–1.07	
75 years old	2	1.09	1.06–1.11	
	5	1.10	1.07–1.13	
	10	1.13	1.05–1.21	

*Note:* This model is stratified by clinical stage.

^a^ Diagnosis age was modeled using a restricted cubic spline due to its non‐linear relationship with survival, and because the age spline violated the proportional hazards assumption, it was also included in an interaction with time. Therefore, hazard ratios for age are shown for various ages (since the hazard associated with an increase in 1 year of age differs by age) and times of follow up (since the hazards are not the same over the follow‐up period). The overall *p* value for the age spline and age spline‐time interaction were 0.002 and 0.009, respectively.

### 3.3 Grade and Stage

**FIGURE 3:**
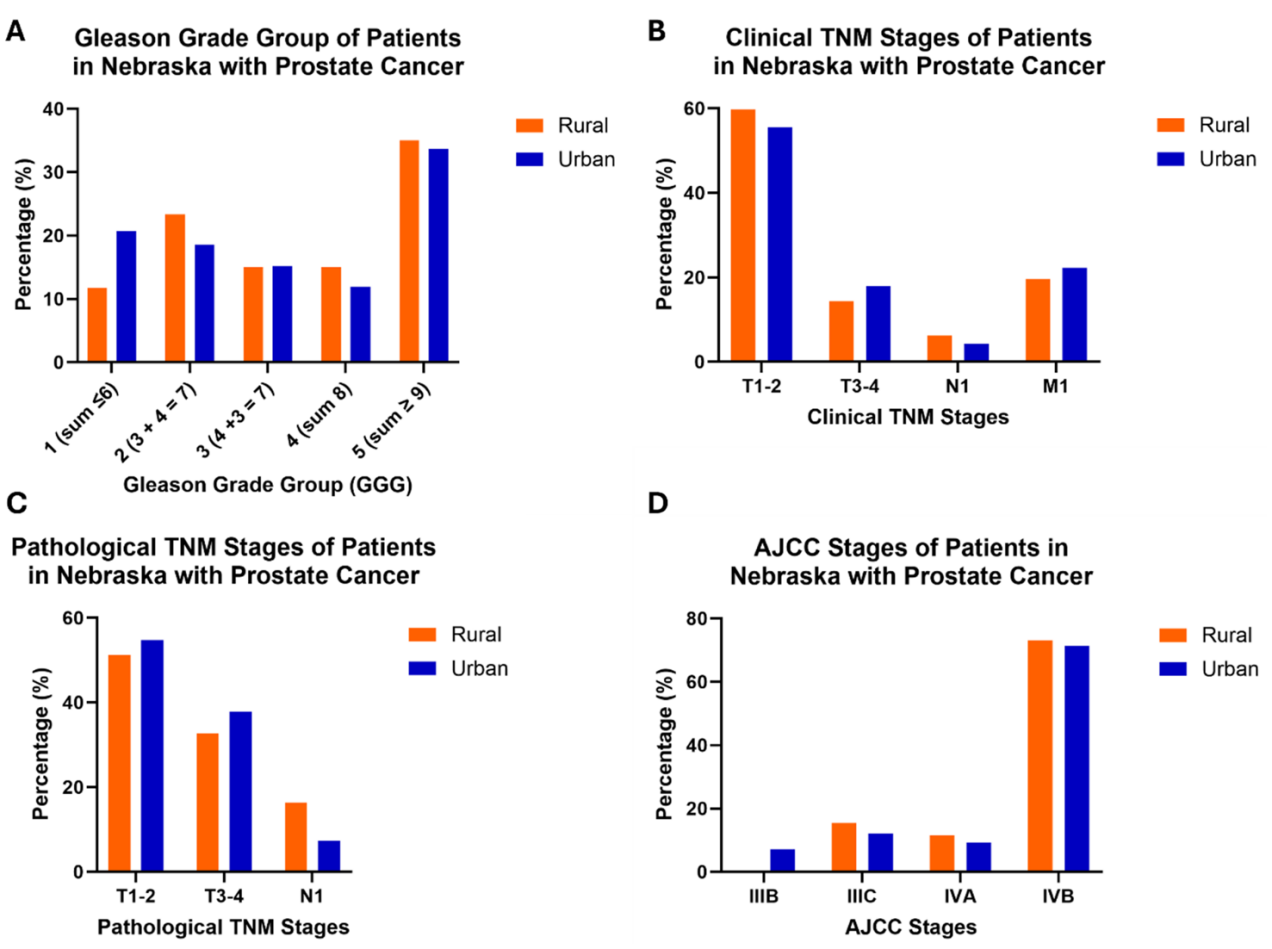
Bar graphs of (A) Gleason grade groups (GGG) (rural *n* = 60, urban *n* = 270), (B) clinical TNM stages (rural *n* = 97, urban *n* = 449), (C) pathological TNM stages (rural *n* = 43, urban *n* = 124), and (D) AJCC stages (rural *n* = 26, urban *n* = 140) of rural (orange) and urban (blue) patients in Nebraska with prostate cancer. Interestingly, rural patients in Nebraska with prostate cancer less frequently had (A) GGG 1 and (D) AJCC stage IIIB and more often had (C) lymph nodes positive for cancer in pathological TNM staging compared to urban patients in Nebraska with prostate cancer.

Prostate cancer grade was analyzed using the International Society of Urologic Pathology (ISUP) Gleason Grade Groups (GGG), based on the Gleason scoring system. Results are shown in Figure 3A and Table 3. Although rural and urban men with prostate cancer did not have statistically significant differences in GGG (*p* = 0.54), there was a lower percentage of rural patients (11.7%) with GGG 1, defined as Gleason score sum 6 or less, compared to urban patients (20.7%).

**TABLE 3:** Clinical characteristics of Nebraskans with prostate cancer in the iCaRe2 registry.

	Urban (*n* = 621)	Rural (*n* = 144)	*p*
Gleason Grade Group	*n* = 270	*n* = 60	0.54^a^
1 (Gleason score sum ≤ 6)	56 (20.7%)	7 (11.7%)	
2 (Gleason score 3 + 4 = 7)	50 (18.5%)	14 (23.3%)	
3 (Gleason score 4 + 3 = 7)	41 (15.2%)	9 (15%)	
4 (Gleason score sum 8)	32 (11.9%)	9 (15%)	
5 (Gleason score sum ≥ 9)	91 (33.7%)	21 (35%)	
Unknown	351	84	
Clinical TNM stages	*n* = 449	*n* = 97	0.62^a^
T1‐2	249 (55.5%)	58 (59.8%)	
T3‐4	81 (18.0%)	14 (14.4%)	
N1	19 (4.2%)	6 (6.2%)	
M1	100 (22.3%)	19 (19.6%)	
Unknown	172	47	
Pathological TNM stages	*n* = 124	*n* = 43	0.22^a^
T1‐2	68 (54.8%)	22 (51.2%)	
T3‐4	47 (37.9%)	14 (32.6%)	
N1	9 (7.3%)	7 (16.3%)	
Unknown	497	101	
Clinical and pathological TNM stage match	*n* = 72	*n* = 23	0.52^a^
Yes	52 (72.2%)	15 (65.2%)	
No	20 (27.8%)	8 (34.8%)	
Unknown	549	121	
AJCC stages	*n* = 140	*n* = 26	0.57^b^
IIIB	10 (7.1%)	0 (0.0%)	
IIIC	17 (12.1%)	4 (15.4%)	
IVA	13 (9.3%)	3 (11.5%)	
IVB	100 (71.4%)	19 (73.1%)	
Unknown	481	118	

^a^ Chi‐squared *p* value.

^b^ Fisher's exact *p* value.

Prostate cancer stage was analyzed based on clinical and pathological TNM and AJCC stages. Our analysis revealed no significant differences between rural and urban cohorts for clinical TNM stages (*p* = 0.62; Figure 3B, Table 3). Similarly, pathological TNM stage analysis revealed no significant differences between rural and urban cohorts (*p* = 0.22; Figure 3C, Table 3). Of patients who had both clinical and pathological TNM stages, excluding metastatic patients, 65.2% of rural patients and 72.2% of urban patients remained at the same stage (*p* = 0.52), and some differences in stage were due to upstaging to T3‐4 tumors or positive lymph node status, particularly in rural patients (Table 4). Analysis of AJCC stage demonstrated no statistically significant difference between rural and urban patients (*p* = 0.57; Figure 3D, Table 3). Interestingly, the rural cohort did not have any patients who qualified for the lowest stage analyzed (IIIB), unlike the urban cohort.

**TABLE 4:** Changes in clinical and pathological TNM stages of rural and urban patients in Nebraska with prostate cancer.

	Clinical TNM stage	Pathological TNM stage	Staging change	*n* (%)
Rural (*n* = 23)	T1‐2N0M0	T3‐4N0M0	Upstage	4 (17.4%)
	T1‐2N0M0	N1M0	Upstage	3 (13.0%)
	T3‐4	N1M0	Upstage	1 (4.3%)
Urban (*n* = 72)	T1‐2	T3‐4	Upstage	1 (1.4%)
	T1‐2N0M0	T3‐4	Upstage	1 (1.4%)
	T1‐2N0M0	T3‐4N0M0	Upstage	1 (1.4%)
	T1‐2N0M0	N1M0	Upstage	4 (5.6%)
	T3‐4	N1	Upstage	1 (1.4%)
	T3‐4N0M0	N1M0	Upstage	1 (1.4%)
	T3‐4N0M0	T1‐2	Downstage	1 (1.4%)
	N1M0	T3‐4	Downstage	1 (1.4%)

## 4 Discussion

In this study, we sought to investigate prostate cancer disparity among rural and urban patients in Nebraska from 1991 to 2023 using our multi‐institutional resource, iCaRe2. Most striking from our analysis was a newfound rural–urban disparity in the age of prostate cancer diagnosis and the age of death in patients with prostate cancer, where rural patients were diagnosed and died with prostate cancer at younger ages than urban patients. The difference of 3.1 years in diagnosis and 4.2 years in death is both statistically and clinically meaningful. Despite these important distinctions in populations, our OS analysis was not statistically or meaningfully significant between the two communities, which is consistent with other analyses of rural–urban prostate cancer disparity [1, 8, 13]. After adjusting for clinical TNM stage and age, the risk of death for rural patients still did not significantly differ from urban patients. Given these results, we posit traditional cancer analyses that focus on OS, which merely captures the time between diagnosis and death, may not be sufficient to capture cancer differences in populations.

In our analysis, RUCA primary codes 4–10 were categorized as rural, which is consistent with the categorization used by the Health Resources and Services Administration (HRSA), to acknowledge that even micropolitan areas in Nebraska are embedded in vast rural regions that highly impact these small communities. Using these definitions, our analysis included 18.8% rural residents, which still underestimates the proportion of rural residents in Nebraska [19]. Urban areas had expectedly more racially and ethnically diverse patients than rural areas, but both patient populations were still predominantly non‐Hispanic White men (> 80%). Racial/ethnic disparities in cancer exist. Black Americans have a higher incidence rate and mortality rate of prostate cancer compared to non‐Hispanic White Americans [22, 23, 24, 25, 26, 27, 28]. One study reported that Alaskan Natives and American Indians also have higher prostate cancer mortality rates compared to non‐Hispanic White Americans [24]. Our study supports the notion that rural residents constitute another distinct group experiencing high rates of adverse outcomes from prostate cancer and warrant further investigation.

Previous national studies have shown rural communities have higher incidences of tobacco use and tobacco‐related cancers compared to their urban counterparts [4, 5, 29] and smoking may impact prostate cancer incidence and risk of death [30]. Therefore, we also investigated smoking status in our analysis. Smoking status was missing from most patients in both communities (> 70%). The available data revealed similar smoking statuses between the two populations, with a majority being non‐smokers (> 50%). Voluntary response bias may be a factor, especially in a database not specifically designed to collect detailed tobacco use information.

Our analyses of rural–urban differences in education, marital status, household income, and employment status did not reveal statistically significant differences. Education and household income are known to be different among rural and urban populations: fewer people in rural communities complete a bachelor's degree and above [31], and those in rural communities often have lower incomes [32]. Our results, though not statistically significant, appear to agree with this trend. Although there was an observed, not statistically significant difference regarding current employment status, where more urban residents were retired than rural residents, this could be related to differences in age of diagnosis, as the presence of prostate cancer prompted the enrollment into the iCaRe2 registry, and we found urban men with prostate cancer were diagnosed at older ages compared to rural men.

We investigated prostate cancer grade through Gleason scores and GGG and stage through clinical and pathological TNM stages and AJCC stages. GGG, clinical and pathological TNM stages, and AJCC stages were not statistically significant for differences between rural and urban patients with prostate cancer but demonstrated some evidence that rural populations may less commonly have prostate cancers with low grade and low stage. Our AJCC stage analysis, which considered Gleason scores and clinical TNM stage, potentially suggested rural patients with prostate cancer in Nebraska may less frequently have the lowest stage in our analysis (IIIB), which may be a consequence of high Gleason scores and positive lymph node status. This would suggest that rural patients may have more clinically aggressive prostate cancer at the time of diagnosis compared to urban patients, which is an important area for future research. However, our analysis of AJCC stage by rural–urban status was not statistically significant and was limited by a lack of initial PSA levels, which precluded analysis of stages lower than IIIB.

Analysis of pathological TNM stage, which also did not show statistically significant differences, potentially suggested rural patients with prostate cancer in Nebraska may be more frequently upstaged to T3‐4, which identifies local cancer growth outside the prostate, and have positive lymph nodes than their urban counterparts. As surgical specimens are required to support pathological TNM staging, one area of future investigation is whether surgery, which is frequently performed in otherwise healthy individuals, is being pursued as treatment over radiation therapy more commonly in rural patients.

Taken together, the current analysis demonstrates disparate clinical presentations between rural and urban patients in that rural men may be diagnosed and die with prostate cancer at younger ages than urban men. Although our analysis of grade and stage was not statistically significant, the earlier onset of prostate cancer as well as the trend of higher grade and stage in rural patients supports the need to investigate potential biological differences in prostate cancer between rural and urban patients. It is known that residents in Nebraska have different environmental influences compared to urban residents that could impact human biology, particularly in relation to pesticide exposure [33, 34]. The positive association between pesticide exposure and aggressive prostate cancer is already known [15, 16, 17, 18]. Air pollution has also been associated with increased prostate cancer risk [35, 36, 37, 38]. Studies on air pollution in rural and urban spaces have demonstrated differences in the quality and quantity of particulate matter sources [39], with particulates associated with agricultural activity and windblown dust being high in the rural US Midwest Great Plains region [40]. However, the differences of these exposures in relation to prostate cancer risk and biology are unknown. Tobacco use, which is more prevalent in rural populations and in the Midwest [41, 42], may be another contributing factor that has known positive associations with aggressive prostate cancer [30, 43]. Other evidence suggests a positive association between heavy alcohol use and aggressive prostate cancer [44, 45]. Although differences in alcohol use among rural and urban communities are a complex topic that requires further investigation [41, 46, 47], rates of heavy alcohol use appear to be higher in rural compared to urban residents in Nebraska [48, 49].

In recognition that different rural areas in the US may be exposed to diverse environmental exposures that impact biology in distinct ways, there is a great need for state‐specific or region‐specific analyses to properly assess rural–urban disparities in the US. A multi‐cancer analysis from the University of Kansas Medical Center shares this conclusion for investigating rural–urban cancer disparities at the local level [50]. Many studies on rural–urban disparities have been conducted using the SEER database, but one rural–urban prostate cancer analysis has discussed the underrepresentation of rural populations in SEER due to the composition of SEER registries in mostly metropolitan areas as a limitation of the resource [14]. We encourage other institutions to focus on rural–urban analyses that can better represent local rural communities and uncover cancer disparities specific to their state or region.

Our study has several important strengths. First and foremost, our study has revealed that there is a clinical prostate cancer disparity among rural men, which has not yet been demonstrated in the field. We focused our investigation on clinically important details that have not yet been well studied in examining rural–urban differences in prostate cancer: age of diagnosis, age of death, GGG, clinical and pathological TNM stages, and AJCC stage. Equally important is our use of a patient registry focused on rural and urban communities in the central US great plains region, iCaRe2, to perform a state‐specific analysis. Consequently, our study contributes a unique and important perspective that encourages further research in rural–urban prostate cancer disparity regarding regional characteristics, clinical presentation, and biological behavior. Potential causes underlying these differences were also discussed and provide a guidepost for future investigations. The iCaRe2 tissue bank provides a unique platform tailored to support bioanalytical components of such investigations.

This study has several limitations. The most important one relates to the large percentage of patients for which data is not available, as documented throughout this study. Further, we recognize this study underrepresents the rural population of Nebraska. Moreover, because iCaRe2 relies heavily on rural patients who are referred to the Fred & Pamela Buffett Cancer Center, which is an urban center, this data may be subject to referral bias. Also, while our analyses suggest potential biological differences in prostate cancer between rural and urban populations, concrete biological evidence is lacking. In addition, the generalizability of our findings to nearby states or the Midwest is unknown, though likely. Finally, there are known associations between rurality and socioeconomic status [3, 51], and socioeconomic status could be a confounding factor in our analysis. These limiting factors are not unique to the current study. In fact, the limiting factors of this study constitute key findings that highlight the large and fundamental knowledge gaps that exist in seeking to understand medical conditions in sparsely populated remote regions in the US. As a scientific community invested in public health, we can strive to better capture this important underserved population.

## 5 Conclusions

In our study, we sought to determine rural–urban cancer disparity among patients in Nebraska diagnosed with prostate cancer from 1991 to 2023. Using our multi‐institutional patient registry, iCaRe2, we investigated race and ethnicity, smoking status, education, marital status, household income, current employment status, age of diagnosis, age of death, overall survival, Gleason scores, clinical and pathological TNM staging, and AJCC staging (stages IIIB–IVB). From this analysis, we determined rural patients with prostate cancer in Nebraska are diagnosed with prostate cancer and die at younger ages than urban patients. Overall survival was similar between the two cohorts, which is consistent with published reports. Investigation into prostate cancer grade and stage was limited by missing information for many patients we analyzed, but the overall clinical characteristics potentially suggested rural patients may have biologically different prostate cancer that is more clinically aggressive than their urban counterparts. Smoking status, education, marital status, household income, and current employment status were similar between the two cohorts. Urban patients were slightly more diverse than rural patients, but non‐Hispanic White men were predominant by a high margin in both populations. Limitations to our study include missing information in our patient registry, not accurately capturing the rural population (including referral bias), unknown generalizability of our findings, and socioeconomic status being a potential confounding variable.

Our results support future investigations into rural–urban cancer disparity. Particularly, we assert that studying rural–urban cancer disparity needs more novel approaches. Exploring clinical features of cancer disease course, such as age of diagnosis and age of death, may better capture differences in cancer behavior, and performing state‐ or region‐specific analyses may better account for unique environmental factors and influences. Moreover, genetic and physiologic studies of tumors from rural and urban patients might identify biological differences in cancers. Overall, we believe rural–urban cancer disparities exist in clinically meaningful ways, and our study contributes to strategies in which we may be able to delineate differences.

## Author Contributions

**Cassie Liu:** conceptualization, investigation, writing – original draft, writing – review and editing, methodology, visualization, project administration. **Kaeli K. Samson:** investigation, methodology, formal analysis, writing – review and editing, visualization. **Oleg Shats:** data curation, writing – review and editing. **Raymond Bergan:** conceptualization, supervision, funding acquisition, writing – review and editing, project administration.

## Ethics Statement

This project did not constitute human subject research as defined by 45CFR46.102 and, therefore, did not require IRB review.

## Conflicts of Interest

The authors declare no conflicts of interest.

## Supporting information


Data S1.

## Data Availability

Datasets generated and/or analyzed during the current study are not publicly available but are available from the corresponding author upon reasonable request.
