# Supplementary material for: Disparities Between Rural and Urban Patients With Prostate Cancer in Nebraska
Source: Cancer Med. 2025 Mar 24;14(6):e70812. doi: 10.1002/cam4.70812 (PMC11931398; doi:10.1002/cam4.70812)
Supplement: Supplementary file 1 — Data S1. [file CAM4-14-e70812-s001.docx]

Supplementary Table 1: Disaggregated Clinical TNM Stages of Rural and Urban Patients in Nebraska with Prostate Cancer

| Clinical TNM Stages | Urban (n = 449) | Rural (n = 97) |
| --- | --- | --- |
| T1-2 | 77 (17.5) | 20 (20.6%) |
| T1-2N0M0 | 172 (38.3%) | 38 (39.2%) |
| T3-4 | 32 (7.1%) | 4 (4.1%) |
| T3-4N0M0 | 49 (10.9%) | 10 (10.3%) |
| N1 | 6 (1.3%) | 3 (3.1%) |
| N1M0 | 13 (2.9%) | 3 (3.1%) |
| M1 | 9 (2.0%) | 2 (2.1%) |
| M1a | 8 (1.8%) | 2 (2.1%) |
| M1b | 77 (17.1%) | 12 (12.4%) |
| M1c | 6 (1.3%) | 3 (3.1%) |

Supplementary Table 2: Disaggregated Pathological TNM Stages of Rural and Urban Patients in Nebraska with Prostate Cancer

| Pathological TNM Stages | Urban (n = 124) | Rural (n = 43) |
| --- | --- | --- |
| T1-2 | 31.5% (39) | 23.3% (10) |
| T1-2N0M0 | 23.4% (29) | 27.9% (12) |
| T3-4 | 16.9% (21) | 16.3% (7) |
| T3-4N0M0 | 21.0% (26) | 16.3% (7) |
| N1 | 2.4% (3) | 2.3% (1) |
| N1M0 | 4.8% (6) | 14.0% (6) |
